# Robust Adaptive Principal Component Analysis Based on Intergraph Matrix for Medical Image Registration

**DOI:** 10.1155/2015/829528

**Published:** 2015-04-19

**Authors:** Chengcai Leng, Jinjun Xiao, Min Li, Haipeng Zhang

**Affiliations:** ^1^Key Laboratory of Nondestructive Testing, Ministry of Education, School of Mathematics and Information Science, Nanchang Hangkong University, Nanchang 330063, China; ^2^State Key Laboratory of Management and Control for Complex Systems, Institute of Automation, Chinese Academy of Sciences, Beijing 100190, China; ^3^Information Center, Jiangxi School of Electronics and Information Engineering, Nanchang 330096, China; ^4^School of Material Science and Engineering, Nanchang Hangkong University, Nanchang 330063, China

## Abstract

This paper proposes a novel robust adaptive principal component analysis (RAPCA) method based on intergraph matrix for image registration in order to improve robustness and real-time performance. The contributions can be divided into three parts. Firstly, a novel RAPCA method is developed to capture the common structure patterns based on intergraph matrix of the objects. Secondly, the robust similarity measure is proposed based on adaptive principal component. Finally, the robust registration algorithm is derived based on the RAPCA. The experimental results show that the proposed method is very effective in capturing the common structure patterns for image registration on real-world images.

## 1. Introduction

Image registration is a fundamental task in medical image processing and has been widely used in multimodal image fusion and tumor detection. Generally speaking, image registration methods can be classified into two categories: area-based and feature-based methods [1]. Area-based methods deal with the images without detecting salient features and adopt optimization algorithms. Mutual information (MI) is one of the most frequently used techniques in area-based methods because of its ability to measure the similarity of the pixels between the reference image and the sensed image [2, 3]. Rivaz et al. [4] proposed an efficient registration method by using Contextual Conditioned Mutual Information (CoCoMI) as the similarity measure in a regularized cost function with a B-spline deformation field and efficiently optimized the cost function by a stochastic gradient descent method, but CoCoMI does not significantly change the registration time. These methods also have some intrinsic limitations due to the intensity distribution, varying illumination, and geometric deformations and are caused, for instance, by noise. Feature-based methods directly use salient features extracted from two images, which is more suitable for illuminated change and complicated geometric deformation. Therefore, comparing with area-based methods, feature-based methods have also been widely used in remote sensing registration [5–7].

Feature-based image registration methods consist of four steps [8]. Among them, the most difficult part of a registration process is the determination of the correspondence between two given feature point sets of the images to be registered. If some correspondences are incorrect, they will produce an incorrect transformation function, which could yield totally wrong results. Therefore, the correct feature correspondences are a key problem for accurate image registration. Graph spectral theory is a powerful tool, which characterizes the global structural properties of graphs using the eigenvalues and eigenvectors of either the adjacency matrix or the closely related Laplacian matrix [9]. Therefore, graph spectral methods have been widely used in computer vision fields for feature matching, such as graph matching [10–14]. Scott and Longuet-Higgins [10] first introduced a Gaussian weighted function to build an interimage proximity matrix to get correspondences from the strength matrix based on singular value decomposition, which is sensitive to the degree of rotation. To resolve this problem, Shapiro and Brady [11] constructed an intraimage proximity matrix for the individual point sets being matched, which aims to capture relational image structures but a sign correction stage is necessary. Carcassoni and Hancock [12] have shown how the modal structure of point sets can be embedded into the framework of the expectation maximization (EM) algorithm and improved the accuracy of correspondences.

Graph spectral method is effective in characterizing the global structure of image, and kernel principal component analysis (KPCA) [7, 15, 16] has a close relationship with graph spectral method, which also has the similar merits and is effective for pattern recognition, regression analysis, and nonparametric estimation. But in order to improve the real-time performance, the dimensionality reduction becomes a necessity. One of the most popular dimensionality reduction algorithms may be principal component analysis (PCA) [15], which performs dimensionality reduction by projecting the original *n*-dimensional data into the *r*-dimensional linear subspace by the leading eigenvectors of the data's covariance matrix. Caelli and Kosinov [17] have extended the Shapiro and Brady method of seeking correspondences by searching for matching that maximize the inner product of the truncated and renormalized eigenvectors. Xu and King [18] exploited the PCA algorithm, which can be used to quickly calculate the approximate matching error of two attributed graphs, and reduce the execution complexity.

Although many researchers have applied the PCA into computer vision and pattern recognition, how to choose adaptively the principle component based on the theorem is still not answered in detail. In this paper, we will give an error analysis theorem and show how to choose adaptively the principal component based on error analysis theorem, which can further extend and improve the theory of PCA. Therefore, we exploit error analysis theorem and propose a novel robust adaptive principal component analysis (RAPCA) method based on intergraph matrix of the objects for image registration. Firstly, the RAPCA is extracted to capture the common structure pattern based on intergraph matrix. Secondly, the robust similarity measure is proposed based on adaptive principal component by projecting both the reference image and the sensed image into the same lower dimensional feature space. Thirdly, the robust registration algorithm is derived based on the RAPCA. The experimental results show that the proposed method is very effective for image registration on real-world images.

This paper is organized as follows.  Section 2 gives the error analysis theorem and a novel image registration algorithm based on RAPCA is proposed.  Section 3 reports the experimental results and we draw the conclusion in Section 4.

## 2. Robust Adaptive Principal Component Analysis Based on Error Analysis Theorem

### 2.1. Error Analysis Theorem

An adaptive principal component analysis based on error analysis theorem is exploited to extract principal feature component to describe the original data and abandon the interference of small eigenvalues corresponding to eigenvectors. We propose error analysis theorem according to Karhunen-Loeve Transform (KLT) [19, 20] and give a quantitative and qualitative analysis to show how to choose adaptively the principal component to avoid choosing the principal component *r* by trial and error.

**Theorem 1 (error analysis theorem).**

Let image (matrix) *X* represent an *N* × *M* matrix, and *X* is transformed into Φ*X* by orthogonal transform Φ; that is, *Y* = Φ*X*; then mean square error can be expressed as follows:

(1) $$\begin{array}{c} \varepsilon \left(\;r\;\right)=\overset{N}{\underset{i=r+1}{\sum }}\phi_{i}^{T}\left[\;C_{X}\;\right]\phi_{i}=\overset{N}{\underset{i=r+1}{\sum }}\lambda_{i}, \end{array}$$

where $\Phi ={\left(\phi_{1}\begin{array}{c} \phi_{2} \end{array}\cdots \phi_{N}\right)}^{T}$ is the orthogonal matrix, $C_{X}=E[(X-\bar{X}){\left(X-\bar{X}\right)}^{T}]$ is the covariance matrix, and *ϕ*_*i*_ is the eigenvector corresponding to the *i*th eigenvalue *λ*_*i*_ of the covariance matrix of *C*_*X*_.

**Proof**

Let *Y* = Φ*X* be the transformed image (matrix) giving an orthogonal transform *X*. The original image (matrix) can be rewritten as

(2) $$\begin{array}{c} X=\Phi^{T}Y=\left(\;\phi_{1}\phi_{2}\cdots \phi_{N}\;\right)\left(\begin{array}{c} y_{1}\\ y_{2}\\ \vdots \\ y_{N} \end{array}\right)=\overset{N}{\underset{i=1}{\sum }}\phi_{i}y_{i}. \end{array}$$

The estimate of *X* can be written as follows:

(3) $$\begin{array}{c} \hat{X}\left(\;r\;\right)=\overset{r}{\underset{i=1}{\sum }}y_{i}\phi_{i}+\overset{N}{\underset{i=r+1}{\sum }}\alpha_{i}\phi_{i}, \end{array}$$

where *α*_*i*_ are undetermined coefficients.

The mean square error is defined as

(4) εrEX−X^rTX−X^r=∑i,j=r+1NEϕiTyi−αiTyj−αjϕj.

We have the following formulation according to orthogonality condition *ϕ*_*i*_^*T*^ *ϕ*_*j*_ = 1, if *i* = *j*; otherwise, *ϕ*_*i*_^*T*^ *ϕ*_*j*_ = 0. Consider

(5) $$\begin{array}{c} \varepsilon \left(\;r\;\right)=\overset{N}{\underset{i=r+1}{\sum }}E\left[\;{\left(\;y_{i}-\alpha_{i}\;\right)}^{2}\;\right]. \end{array}$$

The gradient of the function *ε*(*r*) with respect to *α*_*i*_ is given and let ∂*ε*(*r*)/∂*α*_*i*_ = 0. Thus

(6) $$\begin{array}{c} \alpha_{i}=E\left[\;y_{i}\;\right]=E\left[\;\phi_{i}^{T}X\;\right]=\phi_{i}^{T}\bar{X}. \end{array}$$

Substituting (6) into (5), we have

(7) εr∑i=r+1NEyi−αiyi−αiT=∑i=r+1NϕiTEX−X¯X−X¯Tϕi=∑i=r+1NϕiTCXϕi.

Consequently, we will prove the following formula:

(8) $$\begin{array}{c} \varepsilon \left(\;r\;\right)=\overset{N}{\underset{i=M+1}{\sum }}\lambda_{i}. \end{array}$$

Firstly, we construct an auxiliary function

(9) Lrεr−∑i=r+1NλiϕiTϕi−1=∑i=r+1NϕiTCXϕi−λiϕiTϕi−1.

The gradient of the function *L*(*r*) with respect to *ϕ*_*i*_ is given and let ∂*L*(*r*)/∂*ϕ*_*i*_ = 0. Thus

(10) $$\begin{array}{c} \left[\;C_{X}\;\right]\phi_{i}=\lambda_{i}\phi_{i}. \end{array}$$

From (10), we can see that *λ*_*i*_ is the eigenvalue of covariance matrix *C*_*X*_, and *ϕ*_*i*_ is the corresponding eigenvector. Therefore

(11) $$\begin{array}{c} \varepsilon \left(\;r\;\right)=\overset{N}{\underset{i=r+1}{\sum }}\phi_{i}^{T}\left[\;C_{X}\;\right]\phi_{i}=\overset{N}{\underset{i=r+1}{\sum }}\lambda_{i}. \end{array}$$

So we can choose adaptively principal component according to mean square error (1) in order to avoid choosing the principal component *r* by trial and error.

In order to better exploit error analysis theorem, we will give an error rate (ER) as measure criterion to choose adaptively principal component and the error rate is defined as follows:

(12) $$\begin{array}{c} \text{ER}=\frac{\sum_{i=r+1}^{N}\lambda_{i}}{\sum_{i=1}^{N}\lambda_{i}}. \end{array}$$

Let the size of the matrix *X* be a *N* × *M* dimensionality; the matrix *X* can be written as *X* = (*x*_1_, *x*_2_,…, *x*_*M*_). We construct the covariance matrix *C*_*X*_ = (1/*M*)∑_*i*=1_^*M*^(*x*_*i*_ − *μ*_*X*_)(*x*_*i*_ − *μ*_*X*_)^*T*^, where *μ*_*X*_ = (1/*M*)∑_*i*=1_^*M*^ *x*_*i*_ and we also obtain adaptively the *r* most principal component *U*_*X*_ = [*u*_1_, *u*_2_,…, *u*_*r*_] according to error rate (12) based on singular value decomposition.

### 2.2. Registration Algorithm Based on RAPCA

First, we construct an intergraph matrix of the two sets of features and give adaptive principal component based on intergraph matrix to capture the common structure pattern. Then, we can project the intranormalized Laplacian graph matrix into the same lower dimensional feature space to reveal its structure patterns, respectively. The detailed registration algorithm based on RAPCA is described as follows.

**Step 1 .**

Construct an intergraph matrix *X* ∈ *R*_*M*×*M*_ of the two point sets of *G* and *H*, and the *X* is defined as *X*_*ij*_ = *e* ^−*r*_*ij*_^2^/2*σ*^2^^, where *r*_*ij*_^2^ = ‖*G*_*i*_ − *H*_*j*_‖^2^ is the Euclidean distance between the two point sets and the scale parameter *σ* controls the degree of interaction between the two sets of features.

**Step 2 .**

Construct the covariance matrix *C*_*X*_ = (1/*M*)∑_*i*=1_^*M*^(*x*_*i*_ − *μ*_*X*_)(*x*_*i*_ − *μ*_*X*_)^*T*^ based on an intergraph matrix *X*, and then compute its eigenvalues and eigenvectors.

**Step 3 .**

Choose adaptively the principal component *U*_*X*_ = [*u*_1_, *u*_2_,…, *u*_*r*_] according to error rate (12).

**Step 4 .**

Construct intranormalized Laplacian graph matrices *L*_*G*_ and *L*_*H*_ of point sets, and the normalized Laplacian graph matrix *L*_*G*_ is defined as

(13) $$\begin{array}{c} L_{G}\left(\;v_{i},v_{j}\;\right)=\left\{\begin{array}{cc} \frac{-{\left\|v_{i}-v_{j}\right\|}^{2}}{\sqrt{d_{v_{i}}d_{v_{j}}}} & i\neq j\\ 1 & i=j \end{array}\right. \end{array}$$

and *d*_*v*_*i*__ = ∑_*j*=1_^*M*^‖*v*_*i*_ − *v*_*j*_‖^2^. The normalized Laplacian matrix *L*_*H*_ is similarly defined. Please see [9] for details.

**Step 5 .**

Project *L*_*G*_*v*_*i*___ and *L*_*H*_*v*_*j*___ into the same lower dimensional feature space to reveal the structure patterns, which is defined as *P*_*G*_*v*_*i*___ = *U*_*X*_^*T*^ *L*_*G*_*v*_*i*___ and *P*_*H*_*v*_*j*___ = *U*_*X*_^*T*^ *L*_*H*_*v*_*j*___.

**Step 6 .**

Compute the similarity matrix *S*_*ij*_ = ‖*P*_*G*_*v*_*i*___ − *P*_*H*_*v*_*j*___‖_*F*_^2^, where *S*_*ij*_ reflects the similarity between two point sets *G* and *H*. The points *G*_*v*_*i*__ and *H*_*v*_*j*__ having a 1 : 1 correspondence with one another are given based on the elements of *S*_*ij*_ being both the smallest element in its row and the smallest element in its column. Otherwise, it is many-to-one or one-to-many correspondence. Finally, the transformation function is obtained by the matching relationship.

## 3. Experimental Results and Discussion

In this section, we provide some experimental evaluations of registration algorithm based on RAPCA for image registration. We can choose adaptively the principal component *r* according to the error rate when it is less than 10^−1^, and all the experiments have been done on a personal computer using MATLAB R2010a, with Intel(R) Core (TM) CPU 2.53 GHz and 4.00 GB RAM. There are analyses on real-world images which aim to demonstrate that the proposed method is efficient and feasible comparing with Caelli's method (clustering matching method) [17].

**Figure 1:**
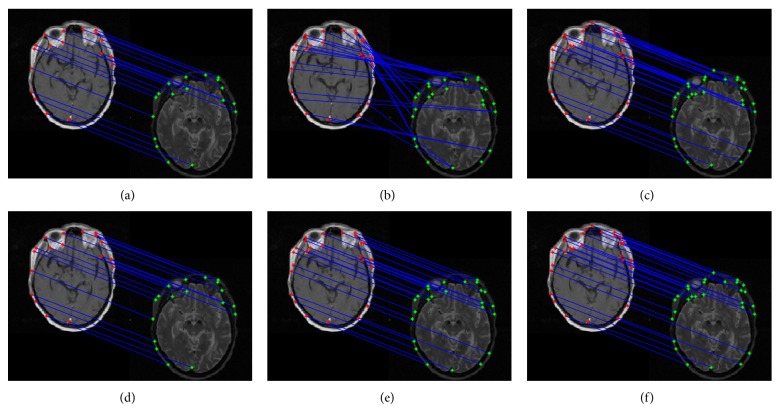
Matching results for different feature points. Top row: matching results based on Caelli's method. Bottom row: matching results based on our method.

To test RAPCA algorithm, we applied it to medical images.  Figure 1 shows the comparison of matching results using our method and Caelli's method [17] to test on T1 and T2 of the 24th slice of a magnetic resonance imaging (MRI) sequence. The 18 feature points, 25 feature points, and 29 feature points are extracted by the Harris Corner Detector [21] from Figures 1(a) and 1(d), 1(b) and 1(e), and 1(c) and 1(f) respectively. From Figures 1(a) and 1(d), we can see that the feature points matching are one-to-one correspondence with Caelli's method and our method. With increase in the number of feature points, Caelli's method produces more many-to-one correspondence as shown in Figures 1(b) and 1(c). However, our method still achieves a one-to-one correspondence as shown in Figures 1(e) and 1(f). Our RAPCA algorithm has high matching ability by projecting intranormalized Laplacian graph matrix into the same lower dimensional feature space based on intergraph matrix, which can reveal the internal geometrical structure information of two point sets. Caelli's method produces some many-to-one correspondence because the distance between some points is very close, which are considered to be in the same class. In addition, Caelli's method is not also stable and can produce different matching results with different feature points extracted. These MRI images are examples to illustrate that the features matching of our method is better than Caelli's method.

**Figure 2:**
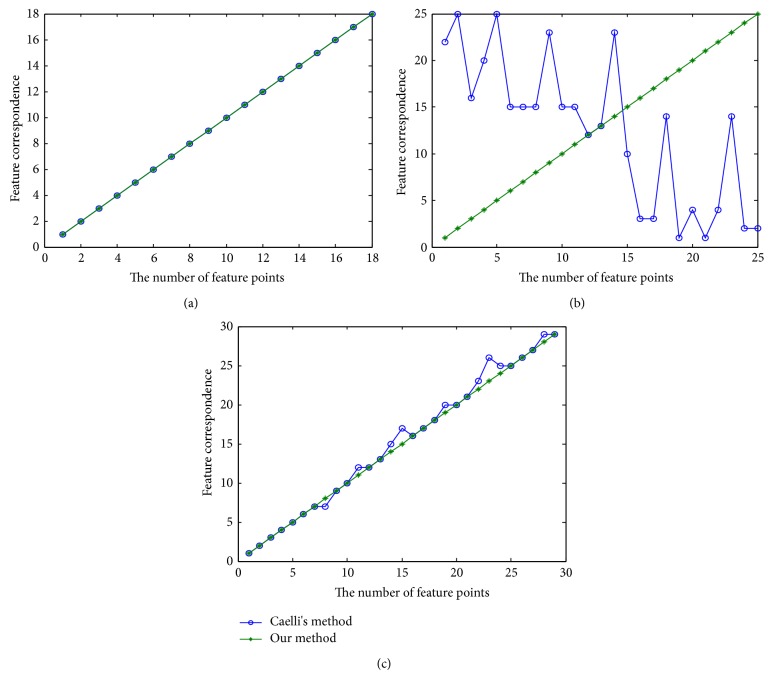
Performance comparison of features correspondence on the MRI images of Caelli's method and our method. (a) Results of Figures 1(a) and 1(d), (b) results of Figures 1(b) and 1(e), and (c) results of Figures 1(c) and 1(f).

Figure 2 shows the matching performance of Caelli's method and our method. From the experimental results, we can see that the feature points correspondences are shown in Figures 2(a), 2(b), and 2(c), which reflect the correct correspondence relationship of Figures 1(a) and 1(d), 1(b) and 1(e), and 1(c) and 1(f), respectively. The results indicate that our method is robust and better than Caelli's method.

**Figure 3:**
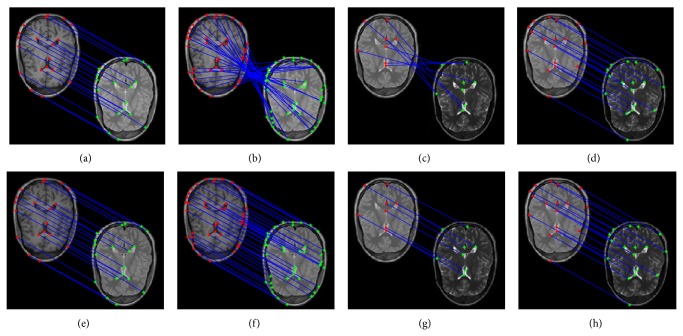
Matching results for different modality images. Top row: matching results based on Caelli's method. Bottom row: matching results based on our method.

To further test our algorithm, we applied the proposed method to the 217 × 181 medical images from the same patient of different modality from the brain datasets [22].  Figure 3 (top row and bottom row) gives matching results for different modality images with Caelli's method and our method, respectively. The first and second column give T1 and PD matching results, and the third and fourth column give PD and T2 matching results, respectively. Figures 3(a) and 3(b) give T1 and PD matching results with different feature points, which produce the different results, and the matching results are bad as shown in Figure 3(b). Figures 3(c) and 3(d) give PD and T2 matching results with different feature points, which also produce the different results, and the matching results are also bad as shown in Figure 3(c). Therefore, Caelli's method is not stable with different feature points from the top row of Figure 3. However, our method can find correct feature correspondences, which show that the proposed method is effective and feasible for different modality images. The reason is that we incorporate the intergraph matrix to capture the common structure pattern and obtain the adaptive principal component based on error analysis theorem. Meanwhile, the robust similarity measure is proposed based on robust principal component by projecting both the reference image and the sensed image into the same lower dimensional feature space to reduce computational complexity.  Table 1 also shows the comparison of computation time which indicates that the computation time of our method is less than Caelli's method.

## 4. Conclusion

In this paper, we present a novel RAPCA method based on intergraph matrix for image registration. Firstly, we give an error analysis theorem, and an adaptive principal component is extracted based on error analysis theorem by incorporating intergraph matrix to capture the common structure pattern of the objects. Secondly, the robust similarity measure is proposed based on robust principal component by projecting both the reference image and the sensed image into the same lower dimensional feature space. Thirdly, the robust registration algorithm is given based on the RAPCA. The experimental results indicate that the proposed method is effective and feasible for image registration.

## Figures and Tables

**Table 1:** Comparison of the computation time of Figures 1 and 3.

Figure and computation time	Caelli's and our method (seconds)			
Figure 1		(a) and (d)	(b) and (e)	(c) and (f)
		0.5608/**0.3639**	0.6268/**0.3834**	0.7122/**0.4057**

Figure 3	(a) and (e)	(b) and (f)	(c) and (g)	(d) and (h)
	0.5071/**0.2931**	0.8037/**0.3573**	0.4036/**0.3030**	0.5259/**0.3670**
